# AI for Prognosis Among People Living With HIV: Protocol for a Systematic Review and Meta-Analysis

**DOI:** 10.2196/91989

**Published:** 2026-08-18

**Authors:** Degninou Yehadji, Markus Hofmann, Petros Isaakidis, Smaila Alidou, Olushina Ayo Junior Ale, Kofivi Mawouko Yena, Kodjo Guidigan, Geraldine Gray, Nadjime Pindra, Gayo Diallo

**Affiliations:** 1 Department of Mathematics, Laboratory of Analysis, Mathematical Modeling and Applications (LAMMA), Faculty of Sciences, University of Lomé, Boulevard Gnassingbe Eyadema, Lomé, Togo, 228 22 21 35 00; 2 Team AHeaD, Bordeaux Population Health Research Center, Inserm 1219, University of Bordeaux, Bordeaux, Nouvelle-Aquitaine, France; 3 Technological University Dublin, Dublin, Leinster, Ireland; 4 Southern Africa Medical Unit, Médecins Sans Frontières, Cape Town, South Africa; 5 Department of Hygiene and Epidemiology, Clinical and Molecular Epidemiology Unit, School of Medicine, University of Ioannina, Ioannina, Epirus, Greece; 6 Department of Public Health, UFR Health Sciences, Université Joseph Ki-Zerbo, Ouagadougou, Burkina Faso; 7 Ministry of Health, Lomé, Togo; 8 Health Data Acumen, Cotonou, Benin; 9 Multi-thematic Clinical Investigation Center Pierre Drouin (CIC-P), Institute for Research and Innovation in Health (IRIS), University of Lorraine, Nancy, France

**Keywords:** AI, machine learning, deep learning, HIV, antiretroviral therapy, ART, prognosis, prediction, outcome, protocol, systematic review, meta-analysis

## Abstract

**Background:**

Advanced HIV disease remains a major global health concern, with nearly 40.8 million people living with HIV as of 2024. Antiretroviral therapy has improved outcomes, but its success depends on timely intervention, adherence, and retention in care. AI, including machine learning and deep learning, offers promising tools for prognostic modeling that could support clinical decision-making and personalized treatment. Existing syntheses, however, have been largely narrative and have not systematically evaluated the performance, risk of bias, reporting quality, or clinical readiness of AI-based prognostic models, leaving a critical gap in understanding their validity and applicability.

**Objective:**

This study aims to conduct a systematic review and meta-analysis of AI-based prognostic models predicting treatment and disease outcomes among people living with HIV, with a focused assessment of predictive performance, methodological rigor, reporting transparency, and potential for clinical implementation.

**Methods:**

A comprehensive search of 5 databases (PubMed, Embase, Scopus, OpenAlex, and IEEE Xplore) covered studies published from January 2015 to December 2025, using a 3-block strategy combining AI, HIV, and clinical outcome terms. Eligible studies are original research using AI to predict individual-level outcomes among people living with HIV. Primary outcomes are virologic and immunologic measures, disease progression, and mortality; secondary outcomes include retention in care, treatment failure, antiretroviral therapy discontinuation, drug resistance, opportunistic infections, hospitalization, and HIV-related comorbidities. Data extraction will use a standardized CHARMS-based form extended with elements from PROBAST+AI, TRIPOD-AI, DECIDE-AI, and the NeurIPS paper checklist; these tools will also inform assessment of risk of bias, reporting transparency, implementation, and reproducibility, with a prespecified framework for integrating overlapping or conflicting judgments. Where studies are clinically and methodologically comparable, accuracy metrics (eg, area under the curve, sensitivity, and specificity) will be synthesized using random-effects models, including bivariate analyses and hierarchical summary receiver operating characteristic curves. Analyses will be conducted in R, with code and study-level data made openly available. Heterogeneity will be explored through subgroup analyses and meta-regression, and the strength of evidence will be graded using an adapted GRADE framework incorporating AI-specific quality dimensions.

**Results:**

The literature search was completed in June 2026, yielding 9194 records across the 5 databases. Following deduplication (3138 records removed), 6056 unique records are pending for title and abstract screening. Full-text eligibility assessment, data extraction, quality appraisal, and quantitative synthesis are expected to begin in August 2026, with final results anticipated for publication by late 2026.

**Conclusions:**

This protocol will provide a focused and methodologically rigorous synthesis of AI-based prognostic models in HIV care, identifying models with robust predictive performance and highlighting critical gaps in validation, reporting, and clinical readiness to inform best practices for future development and implementation.

## Introduction

Advanced HIV disease (AHD), caused by HIV infection, remains a major global health issue [1]. Since the epidemic began, more than 88.4 million people have been infected, and 42.3 million have died from AHD-related illnesses. As of 2024, 40.8 million people were living with HIV, and 630,000 deaths were reported due to AHD [2]. Antiretroviral therapy (ART) is a key strategy for controlling HIV and is recommended for all people living with HIV due to its benefits for prolonging life and reducing transmission. The HIV treatment cascade outlines 5 stages: diagnosis, linkage to care, retention in care, adherence to ART, and viral suppression [3-5].

ART outcomes include clinical, virologic, and immunologic responses, which are mainly tracked through viral load. Other outcomes include disease progression, opportunistic infections, hospitalizations, drug resistance, toxicity, organ failure, and mortality. Treatment adherence and retention in care are events of interest that are critical to sustaining positive outcomes [6-10].

In health care, prognosis is critical for informing clinical decisions and improving patient outcomes. Particularly in HIV care, prognosis has traditionally been assessed through statistical methods such as Kaplan-Meier curves and Cox regression models [11,12]. However, with advancements in machine learning (ML), these traditional approaches are increasingly supplemented by AI-based tools that may offer complementary predictive capabilities for treatment outcomes, potentially supporting more individualized decision-making [13-17]. AI includes supervised learning (using labeled data for classification or regression) and unsupervised learning (finding patterns in unlabeled data) [18]. Various ML and deep learning algorithms are applied in health care, including logistic regression, support vector machines, k-nearest neighbors, decision trees, random forest, gradient boosting machines, adaptive boosting, extreme gradient boosting, light gradient boosting machine, categorical boosting, and naive Bayes, as well as neural networks such as convolutional neural networks, recurrent neural networks (RNNs), long short-term memory (LSTM) networks, and multilayer perceptrons [19-22].

AI applications are transforming the way health care systems handle vast amounts of data, from electronic health records to medical imaging and genomic data. These technologies have shown potential to improve predictive capabilities in some settings, including resource-limited environments where traditional methods may fall short [23-26]. In HIV care, AI models can predict a range of outcomes, including diagnostic results, retention in care, and viral suppression, and may offer incremental value in supporting personalized ART and care pathway optimization [27]. In addition to static models trained on baseline predictors, a growing body of work applies dynamic prediction frameworks such as RNNs, LSTM networks, and landmark models that continuously update prognostic estimates as new longitudinal data become available, enabling real-time prediction of both whether and when a clinical event may occur.

Despite its promise, AI in health care faces challenges, including data quality, model transparency, interpretability, and ethical concerns such as privacy and bias [28]. A recent global systematic review by Ngcobo et al [29] synthesized the rapidly expanding literature on AI applications across the HIV care continuum, encompassing diagnostics, testing, retention in care, treatment monitoring, and patient support. However, that review adopted a broad, narrative mapping approach and did not focus specifically on AI-based prognostic prediction models, nor did it quantitatively evaluate model performance, assess risk of bias (RoB) using prediction-specific tools, or examine reporting quality and readiness for clinical implementation.

Consequently, a critical gap remains in the systematic evaluation of AI-based prognostic models developed for people living with HIV, particularly with respect to their predictive performance, methodological robustness, reporting transparency, and applicability in clinical settings. To address this gap, the present study aims to conduct a systematic review and meta-analysis of AI-based prognostic models predicting treatment and disease outcomes among people living with HIV, with a focused assessment of model performance, RoB, reporting quality, and clinical applicability.

## Methods

### Study Registration and Reporting Guidelines

This systematic review and meta-analysis is registered with the International Prospective Register of Systematic Reviews (PROSPERO: CRD420251034551). The protocol was developed following the PRISMA-P 2015 (Preferred Reporting Items for Systematic Review and Meta-Analysis Protocols) guidelines (Checklist 1) and the results will be reported according to the TRIPOD-SRMA (Transparent Reporting of Multivariable Prediction Models for Individual Prognosis or Diagnosis: Checklist for Systematic Reviews and Meta-Analyses) guidelines [30,31].

### Research Questions

The research questions are structured according to the prediction model life cycle and reporting standards for prognostic model systematic reviews, in alignment with the TRIPOD-SRMA and PROBAST+AI (Prediction Model Risk of Bias Assessment Tool Extension for AI) guidelines . The questions are designed to evaluate model purpose, development characteristics, validation approaches, predictive performance, RoB, and potential clinical applicability (Table 1).

**Table 1.** Research questions for the systematic review and meta-analysis of studies on AI for prognosis among people living with HIV.

Methodological domain	Review question
Model purpose and prognostic scope	What prognostic outcomes are AI-based models designed to predict among people living with HIV?
Model development characteristics	What types of AI algorithms are used to develop prognostic models for people living with HIV? What candidate predictor variables are included, and how are they selected or engineered? What data sources and data types are used for model development?
Model validation and generalizability	What internal validation methods and external validation strategies are used? To what extent are models validated across different populations, settings, or time periods?
Predictive performance and calibration	What measures of discrimination (eg, AUC^a^, sensitivity, and specificity) and calibration (eg, calibration slope and observed-to-expected ratio) are reported? How does predictive performance vary by outcome type, model class, and validation approach?
Risk of bias and reporting quality	What is the risk of bias across PROBAST+AI^b^ domains (participants, predictors, outcomes, and analysis)? To what extent do included studies adhere to TRIPOD-AI^c^ reporting recommendations?
Clinical applicability and implementation readiness	What evidence is reported regarding model interpretability, clinical usability, and integration into HIV care pathways? What barriers to implementation are identified?

a AUC: area under the curve.

b PROBAST+AI: Prediction Model Risk of Bias Assessment Tool Extension for AI.

c TRIPOD-AI: Transparent Reporting of Multivariable Prediction Models for Individual Prognosis or Diagnosis Extension for AI.

### Outcomes of Interest

To guide data extraction and analysis, a predefined list of potential outcomes of interest was developed based on current HIV care guidelines, clinical practice, and previous literature. These outcomes reflect key aspects of disease progression, treatment response, and patient engagement across the continuum of care [4].

Outcomes are categorized as primary prognostic outcomes or secondary prognostic outcomes, reflecting their clinical relevance, temporal relationship to prognosis, and suitability for predictive modeling and quantitative synthesis. Primary prognostic outcomes are restricted to end points that most directly and proximally reflect HIV disease progression and ART effectiveness, and that are most consistently defined across international guidelines and the existing literature. These comprise virologic outcomes (viral suppression and virologic failure), immunologic outcomes (cluster of differentiation 4 [CD4] count trajectory and immunologic failure), progression to AHD, and mortality (all-cause and HIV-related). These 4 outcome domains represent the core prognostic targets of this review and will be prioritized in both narrative synthesis and, where the prespecified eligibility criteria for pooling are met, quantitative meta-analysis.

Secondary prognostic outcomes include clinically relevant end points that indirectly influence long-term prognosis but are characterized by greater heterogeneity in definitions, measurement approaches, and clinical interpretation across studies. These include retention in care (including time to loss to follow-up), treatment failure, ART discontinuation, drug resistance, opportunistic infections, hospitalization, and HIV-related comorbidities, and they will be included in the narrative synthesis but not prioritized for quantitative pooling. Their secondary designation reflects methodological considerations rather than a judgment on their clinical importance.

Outcomes primarily related to diagnostic performance, testing uptake, chatbot engagement, or knowledge and behavioral change are not considered primary end points and are excluded from the main outcome framework, as they fall outside the scope of individual-level prognostic prediction. This structured outcome classification enables targeted synthesis of AI-based prognostic models and supports stratified evaluation of model performance, RoB, and clinical applicability. All identified outcomes will be included in the narrative systematic review regardless of the number of studies available. Meta-analysis will be reserved for outcomes for which a minimum of 10 studies reporting comparable performance metrics are identified, in line with established recommendations for meta-analysis of prediction model performance [32,33]. For outcomes below this threshold, findings will be synthesized narratively. Table 2 summarizes the prognostic outcome categories and representative end points included in this review.

**Table 2.** Prognostic outcomes of interest for AI-based models among people living with HIV: categories, specific outcomes, and operational definitions.

Outcome category and specific outcome	Operational definition
Primary prognostic outcomes	
Virologic outcomes	
Viral suppression	HIV RNA below a defined threshold, commonly <200 copies/mL, sustained over a specified period
Virologic failure	HIV RNA above a defined threshold (commonly ≥200 or ≥1000 copies/mL) after at least 6 months on ART^a^
Virologic rebound	Return of detectable HIV RNA after confirmed viral suppression
Time-to-virologic failure	Time from ART initiation or viral suppression to confirmed virologic failure
Immunologic outcomes	
CD4^b^ count decline	Reduction in absolute CD4 T-cell count below a clinically defined threshold (eg, <200 or <350 cells/mm^3^)
Immunologic failure	Failure to achieve or maintain a CD4 count response despite ART, as per WHO^c^ or study-specific criteria
CD4/CD8 ratio	Change in the CD4/CD8 T-cell ratio as a marker of immune restoration
Disease progression outcomes	
HIV disease progression	Transition to a more advanced WHO clinical stage or to AHD^d^ (CD4 <200 cells/mm³ or WHO stage 3 or 4)
Mortality outcomes	
All-cause mortality	Death from any cause during follow-up
HIV-related mortality	Death directly attributable to HIV disease progression or AIDS-defining illness
Comorbidity-related mortality	Death attributable to a comorbid condition in the context of HIV infection
Secondary prognostic outcomes	
Treatment-related outcomes	
Treatment failure	Composite or individual virologic, immunologic, or clinical failure as defined by study authors or international guidelines
ART discontinuation	Permanent or temporary cessation of ART for any reason, including toxicity, patient decision, or clinical indication
Drug resistance	Emergence of genotypic or phenotypic resistance to one or more antiretroviral drug classes, confirmed by resistance testing
Engagement-related outcomes	
Retention in care	Continued engagement with HIV clinical services over a defined follow-up period, as per study-specific or guideline-based definitions
Loss to follow-up	Absence from HIV care for a defined period, commonly 90 or 180 days after the last scheduled visit, without documented transfer or death
ART adherence	Proportion of prescribed ART doses taken over a defined period, commonly assessed as ≥95% for optimal adherence
Appointment attendance	Attendance at scheduled HIV clinic visits; defaulting defined as missing one or more consecutive appointments without prior notification
Clinical complication outcomes	
Opportunistic infections	Occurrence of a new AIDS-defining or non-AIDS-defining opportunistic infection during follow-up
HIV-related comorbidities	Development of conditions causally or epidemiologically associated with HIV or ART (eg, cardiovascular disease, renal disease, metabolic disorders)
Hospitalization	Unplanned inpatient admission for any HIV-related or ART-related cause during follow-up

a ART: antiretroviral therapy.

b CD4: cluster of differentiation 4.

c WHO: World Health Organization.

d AHD: advanced HIV disease.

The unit of analysis for both narrative synthesis and meta-analysis will be the outcome-model pair. When a study reports multiple models or multiple outcomes, each combination will be treated as a separate analytical unit, subject to the prespecified rules described in the *Statistical Analysis* section.

### Search Strategy

Five bibliographic databases—PubMed, Embase, Scopus, OpenAlex, and IEEE Xplore—were searched using 3 concept blocks: an AI block, an HIV terminology block, and an outcomes block, combined using the Boolean operator AND. The AI block encompasses a broad range of terms covering AI methods, ML, deep learning, supervised learning, decision support, and related contextual terms, including automated systems, digital health, and health informatics. The HIV block covers all commonly used designations for HIV and AIDS. The outcomes block covers the primary and secondary prognostic outcomes of HIV care (Checklist 1; Multimedia Appendix 1). Papers in any language published between January 2015 and December 2025 are included.

In the first instance, 2 investigators will independently screen the titles and abstracts, referring to the inclusion criteria. Any discrepancies will be resolved in consultation with a third investigator. Then, all full-text studies meeting the inclusion criteria will be selected for relevant data extraction. Zotero (Corporation for Digital Scholarship) will be used as the reference manager. Duplicate removal and paper screening will be conducted in Rayyan.

### Study Selection

The selection of studies will follow a structured screening process based on predefined inclusion and exclusion criteria (Table 3). For the purposes of this review, AI-based prognostic models are defined as models that use ML or deep learning algorithms to predict individual-level outcomes. This includes, but is not limited to, decision trees, random forests, gradient boosting machines, support vector machines, and neural networks and their variants [19]. Logistic regression occupies a boundary position: it will be considered eligible when clearly framed and implemented as an ML model within a supervised learning pipeline, but not when applied as a standalone conventional statistical model without such ML elements [34]. Survival analysis methods (eg, Kaplan-Meier method, Cox proportional hazards analysis, and the log-rank test) are considered conventional statistical approaches and will not be eligible as primary models [35].

**Table 3.** Selection criteria for the systematic review and meta-analysis of studies on AI for prognosis among people living with HIV.

Criterion	Inclusion	Exclusion	Rationale
Publication type	Original articles, full preprint manuscripts	Any other sources^a^	To ensure inclusion of original studies
Review type	Peer-reviewed, preprints without peer review	Abstracts without full papers	Full articles, including preprints, provide sufficient methodological and performance details for appraisal, while abstracts alone lack the necessary depth
Publication period	Studies published between January 2015 and December 2025	Studies published before January 2015 or after December 2025	The period from 2015 onwards reflects the marked acceleration in AI and machine learning applications in HIV clinical research [13]
Language	Publications in any language	None	Include potentially relevant studies published in languages other than English
Study objective	Studies focusing on the use of AI to predict outcomes among people living with HIV	Studies focusing on the use of AI in HIV research for purposes other than individual-level prognostic prediction^b^	To ensure the review includes only studies relevant to individual-level prognosis among people living with HIV

a Books, letters, commentaries, review articles, case reports, methodological papers, etc.

b This includes studies aiming to (1) predict outcomes at the cellular, viral, molecular, facility, or administrative level, (2) determine the factors associated with outcomes, (3) determine or optimize treatment, (4) predict screening results or seroconversion, (5) predict patient segmentation, and (6) conduct epidemiological surveillance of seroconversion.

### Data Extraction

A standardized data collection form will be developed and piloted to extract information from all full-text papers included after screening. The structure of this form will be adapted from the CHARMS (Critical Appraisal and Data Extraction for Systematic Reviews of Prediction Modeling Studies) checklist and expanded to integrate elements required for subsequent appraisal using the PROBAST+AI, the TRIPOD-AI (Transparent Reporting of Multivariable Prediction Models for Individual Prognosis or Diagnosis Extension for AI) checklist , the reporting guideline for the DECIDE-AI (Developmental and Exploratory Clinical Investigations of Decision Support Systems Driven by AI), and the conference on NeurIPS (Neural Information Processing Systems) paper checklist [36-41] .

Specifically, the form will capture core study descriptors, including study identification, setting, population characteristics, prognostic outcome, intended use of the model, algorithm type, predictor variables, data sources, preprocessing methods, sample size, validation approaches, and performance metrics across 3 domains: discrimination (eg, area under the curve [AUC], sensitivity, and specificity), calibration (eg, calibration slope, calibration-in-the-large, and observed-to-expected ratio), and clinical use (eg, net benefit and decision curve analysis results).

In addition, fields will be included to document reported bias and limitations (as per CHARMS), domain-level information relevant to PROBAST+AI (participants and data sources, predictors, outcome, and analysis), and TRIPOD-AI and DECIDE-AI reporting items. Reproducibility-specific information will be assessed for each included study using the NeurIPS paper checklist, covering the following: experimental results reproducibility, open access to data and code, experimental settings and hyperparameter reporting, statistical significance of experiments, compute resources disclosure, licensing for existing assets, and documentation of new assets [38].

The extraction will be conducted independently by 2 investigators using the standardized form, with discrepancies resolved through discussion or adjudication by a third investigator. This comprehensive data collection framework is designed to ensure compatibility with downstream critical appraisal tools, facilitate robust quality assessment, and support transparent synthesis of findings across AI-based prognostic models for people living with HIV.

### Appraisal of Studies

The methodological quality, the quality and reproducibility of model development, RoB, applicability of prediction models, transparency and completeness of reporting, and clinical implementation of the selected studies will be assessed using a multitool approach. RoB, quality of model development, and applicability will be evaluated using an adaptation of the combined CHARMS checklist and PROBAST approach, as developed by Fernandez-Felix et al [42], integrating additional aspects included in PROBAST+AI. Two investigators will independently perform these assessments, with disagreements resolved in consultation with a third investigator.

PROBAST+AI is a tool for appraising the quality of model development, RoB, and the applicability of prediction models. Building on PROBAST, it introduces a clear distinction between 2 phases: model development and model evaluation, each assessed separately across 4 key domains (participants and data sources, predictors, outcome, and analysis). The quality of the model development process is assessed using 16 signaling questions across the 4 domains. Responses to each question are scored as “Yes,” “Probably yes,” “No,” “Probably no,” “No information,” or “Not applicable.” Each domain receives a quality judgment rated as low, high, or unclear concern. A model development process is judged to be of high concern regarding quality if any domain is rated as high concern, or of low concern if all domains are rated as low concern. This quality assessment flags whether the model was developed using sound design and analytical practices, such as appropriate handling of missing data, sufficient sample size, proper feature selection, and adequate treatment of class imbalance and overfitting.

To assess RoB in model evaluation, PROBAST+AI uses 18 signaling questions, also across the same 4 domains. This part assesses systematic errors that might distort performance estimates such as calibration, discrimination, and clinical utility. Each domain is rated for RoB as low, high, or unclear. The overall RoB in model evaluation is considered high if any domain is judged to have high RoB, or low only if all domains are rated as low RoB. Applicability concerns are evaluated in the first 3 domains during both the development and evaluation phases. These relate to how well the data, predictors, and outcomes align with the user’s intended use of the model or with the review question. A model is rated as having high concern for applicability if any domain shows a mismatch, or low concern for applicability only if all domains are appropriately aligned. Each study may receive 3 overall ratings: (1) model development quality (low, high, or unclear), (2) model evaluation RoB (low, high, or unclear), and (3) applicability (low, high, or unclear).

To evaluate the transparency and completeness of reporting specific to AI-based prediction models, the TRIPOD-AI checklist will be used. This tool ensures that studies adequately report critical elements, including data sources, model development, validation processes, performance metrics, and AI-specific components such as algorithm rationale and training dynamics [40]. Each reporting item will be assessed as fully reported, partially reported, or not reported. A study will be considered to have low reporting transparency if critical model development or validation elements are partially reported or not reported.

For studies that involve early-stage clinical implementation or real-world testing of AI-driven decision support tools, the reporting guideline for the DECIDE-AI will be applied [39]. This guideline facilitates structured evaluation of system integration, user interactions, contextual influences, and implementation fidelity. Its use will support the identification of barriers and facilitators to effective implementation in clinical environments for people living with HIV. DECIDE-AI organizes its reporting items under themes. Each of the themes will be assessed for reporting completeness—fully reported, partially reported, or not reported.

Finally, each study will be reviewed using a checklist adapted from the NeurIPS paper checklist to assess the replicability of the reported AI models [38]. For each reporting item, studies will be evaluated based on whether the information is available, unavailable, or unclear. A study will be considered to have limited reproducibility if it fails to meet reporting standards across core reproducibility themes.

The rationale for combining 5 appraisal tools is that no single instrument addresses all dimensions relevant to AI-based prognostic model reviews. CHARMS and PROBAST+AI address methodological quality, RoB, and applicability to model development and evaluation. TRIPOD-AI assesses reporting completeness and transparency. DECIDE-AI is applied only to studies involving early-stage clinical implementation and addresses system integration, user interaction, and real-world deployment fidelity—dimensions absent from PROBAST+AI and TRIPOD-AI. The NeurIPS paper checklist addresses computational reproducibility, including code availability, hyperparameter reporting, and licensing, none of which are covered by the clinical appraisal tools. Together, these instruments provide complementary coverage across methodological, reporting, implementation, and reproducibility domains.

To ensure that judgments from the 5 appraisal instruments can be interpreted coherently, an explicit integration framework will govern how domain-level ratings are handled, how overlaps are managed, and how conflicting judgments are reported. As such, each tool will be treated as authoritative within its primary domain. PROBAST+AI is the authoritative instrument for methodological quality, RoB, and applicability to model development and evaluation. TRIPOD-AI is the authoritative instrument for reporting completeness and transparency. DECIDE-AI is applied exclusively to studies involving early-stage clinical implementation and addresses system integration, user interaction, and real-world deployment fidelity. The NeurIPS paper checklist is the authoritative instrument for computational reproducibility, covering code availability, hyperparameter reporting, and licensing. CHARMS provides the structural data extraction framework underpinning the PROBAST+AI appraisal and does not generate independent quality judgments.

When TRIPOD-AI and PROBAST+AI assessments appear to conflict—for instance, where a study is rated as low RoB by PROBAST+AI but has incomplete reporting of critical model development elements by TRIPOD-AI—both ratings will be reported transparently without forced reconciliation, as they reflect genuinely distinct dimensions of study quality. The implications of such discordance for confidence in a study’s findings will be discussed narratively.

An overall per-study appraisal summary table will be produced, presenting domain-level ratings from each applicable tool side by side to enable an overview of patterns of concordance and discordance across instruments. A composite study-level quality flag will be derived from this table using a prespecified rule: a study will be flagged as having substantive quality concerns if it meets any of the following conditions: (1) high or unclear RoB in any PROBAST+AI domain, (2) partial or nonreporting of 2 or more critical TRIPOD-AI items related to model development or validation, or (3) limited reproducibility across 2 or more NeurIPS checklist themes. This composite flag will inform sensitivity analyses and contextualize the narrative synthesis but will not replace the tool-specific domain ratings reported in the results.

### Statistical Analysis

Data extracted into structured spreadsheets will be analyzed using R (R Foundation for Statistical Computing), with meta-analytic models fitted using the metafor package, bivariate and hierarchical summary receiver operating characteristic (HSROC) models fitted using the mada package, and robust variance estimation using the robumeta package. Study characteristics, model features, and outcomes will be summarized using descriptive statistics and visualized in tables and figures, accompanied by a structured narrative synthesis.

Narrative synthesis will constitute the primary analytical approach, given the anticipated heterogeneity in AI model architectures, outcome definitions, predictor variables, validation methods, and reported performance metrics across included studies. Quantitative synthesis will be undertaken only as a secondary, conditional step, restricted to subgroups of studies meeting all of the following prespecified criteria for clinical and methodological comparability: (1) a minimum of 10 studies reporting the same outcome using comparable definitions, (2) sufficient homogeneity in AI algorithm class, (3) use of a comparable validation strategy, (4) reporting of a common performance metric, and (5) absence of dataset overlap. If any of these conditions are not met for a given outcome or subgroup, findings will be synthesized narratively rather than pooled. Furthermore, even where minimum comparability criteria are met, meta-analysis will be suspended, and results will be reverted to narrative synthesis if the *I*² statistic exceeds 75% and no covariate explains the observed heterogeneity [32,33].

When quantitative synthesis is feasible, 2 analytically distinct and mutually exclusive pooling streams will be applied depending on the metric reported. Studies reporting incompatible metrics, such as *F*_1_-score, accuracy, or Matthews correlation coefficient, will not be pooled across metric types and will be retained for narrative synthesis. For models reporting AUC, values will be logit-transformed prior to pooling using inverse-variance weighting under a random-effects model. AUC summarizes discrimination across all possible classification thresholds and is therefore threshold-independent, making this approach appropriate. For binary classification outcomes where studies report paired sensitivity and specificity values at a fixed or study-specific decision threshold, diagnostic test accuracy meta-analysis will be performed using bivariate random-effects models to jointly estimate pooled sensitivity and specificity, with HSROC curves used for visualization [43-47]. Bivariate and HSROC methods are justified because AI-based binary classifiers evaluated at varying decision thresholds across studies exhibit the same statistical structure as diagnostic tests evaluated at varying cut-offs: threshold heterogeneity induces a negative correlation between sensitivity and specificity across studies, which bivariate models clearly account for by jointly modeling the 2 parameters rather than treating them as independent. Pooling them separately would yield misleading estimates and understate uncertainty [48,49]. These methods will be applied only to subgroups reporting paired sensitivity and specificity values and will not be combined with the AUC pooling stream [43,47].

Predictive performance will be evaluated across 3 complementary domains—discrimination, calibration, and clinical utility, with a metric priority hierarchy governing pooling decisions and the handling of incompatible reporting. Discrimination is the primary performance domain. AUC is the primary metric, given its threshold-independence and consistent reporting across AI prediction model studies, and will be logit-transformed and pooled using inverse–variance-weighted random-effects models. Where AUC is not reported, but paired sensitivity and specificity values are available, these constitute the secondary discrimination metrics and will be pooled using bivariate random-effects models and HSROC curves as described earlier. The 2 streams are mutually exclusive and will not be combined. Where a study reports neither AUC nor paired sensitivity and specificity, it will be retained in narrative synthesis but excluded from quantitative pooling, with the reason documented. Where uncertainty measures for the AUC are not reported, standard errors will be estimated from sample size and event counts using established formulae for the logit-transformed AUC [32]. If estimation is not feasible, the study will be excluded from quantitative pooling and retained in narrative synthesis. No imputation of missing performance metrics or uncertainty measures will be performed.

Calibration is the secondary performance domain and will be assessed using the calibration slope, calibration-in-the-large, and observed-to-expected ratios, where reported. Given the anticipated inconsistency in calibration reporting across studies, calibration metrics will be synthesized descriptively in all cases and visualized using calibration plots, where data permit [33,50]. No quantitative pooling of calibration metrics will be undertaken, regardless of the number of studies reporting them, given the high expected heterogeneity in calibration assessment methods and reference thresholds across studies.

Clinical utility is the tertiary performance domain and will be assessed where reported, primarily through decision curve analysis and net benefit metrics [51,52]. These will be summarized narratively, as the anticipated variability in decision thresholds and clinical contexts across studies precludes meaningful quantitative pooling.

Between-study heterogeneity will be assessed using the *I*² statistic and explored visually using forest plots [53]. Prespecified subgroup analyses and meta-regression will be conducted to investigate the sources of heterogeneity, including AI algorithm class, model architecture, predictor variable categories, outcome type, validation approach, and data source. RoB will be integrated into synthesis decisions. Models judged to have a high RoB in any PROBAST+AI domain will be excluded from quantitative pooling with low-risk models and will be analyzed separately.

Four sensitivity analyses have been defined. First, analyses will be repeated excluding studies with high or unclear RoB in any PROBAST+AI domain to assess the influence of methodological quality on pooled estimates. Second, analyses will be stratified by validation strategy to examine the potential optimism of internal vs external validation. Third, analyses will be restricted to studies using consensus-based outcome definitions, to assess the impact of outcome heterogeneity. Fourth, analyses will be repeated excluding studies with suspected overlapping datasets. Any deviation from these prespecified analyses will be documented and justified.

Several rules have been established to handle common complexities in AI prediction model reviews. Where a study reports multiple models predicting the same outcome and reporting the same performance metric, all will be included as separate units of analysis, with adjustments for within-study and within-cohort correlations. Three-level meta-analytic models will be used as the default approach in this situation, partitioning variance at the model-within-study level and the between-study level. Where 3-level model convergence fails, or the number of studies contributing multiple models is insufficient to reliably estimate the within-study variance component, robust variance estimation will be applied as a fallback, using the correlated effects weighting scheme with a working correlation of 0.80, in line with established recommendations for prediction model reviews [32]. Where multiple outcomes are reported, each outcome-model pair will serve as the unit of analysis, assessed separately within predefined subgroups without requiring within-study correlation adjustment across outcomes. Where multiple validation sets are reported, external validation results will be prioritized over internal validation; if multiple external sets are available, the largest or most representative will be selected, or pooled if sufficiently similar, with all results documented. Where studies draw on the same dataset, overlaps will be identified using data source, country, period, and sample size as indicators. Only the most recent or comprehensive study will be included in the quantitative synthesis, with sensitivity analyses conducted and overlaps described narratively [32,33,54].

When studies report both AI-based and conventional prognostic models applied to the same outcome and dataset, model class will be included as a covariate in meta-regression to examine whether discriminative performance systematically differs between approaches; this meta-regression will be restricted to studies providing directly comparable metrics [32,33,50,54].

All statistical analyses will adhere to current best-practice recommendations for prediction model systematic reviews and meta-analyses, ensuring that quantitative synthesis is performed only when methodologically justified and clinically interpretable [40,54].

### Strength of Evidence

The overall strength of the body of evidence across selected studies will be assessed using an adapted framework based on the GRADE (Grading of Recommendations Assessment, Development, and Evaluation) approach, supplemented with AI model-specific considerations [55,56]. This evaluation will focus on the collective confidence in the estimated effects of AI model performance in predicting outcomes among people living with HIV. The assessment will be conducted across standard GRADE domains—RoB, consistency, directness, precision, and publication bias, with additional AI-specific domains derived from the TRIPOD-AI checklist.

RoB will be informed by domain-level evaluations from PROBAST+AI. Downgrading will occur when high or unclear RoB is observed in critical domains (participants, predictors, outcome, and analysis), particularly if prevalent across multiple studies. Consistency will be evaluated based on the degree of heterogeneity in reported model performance metrics (eg, AUC, sensitivity, and specificity), quantified using the *I*² statistic and assessed visually through forest plots. Directness pertains to how well the included studies align with the target population (people living with HIV), the prognostic outcomes of interest, and the intended clinical applications of AI models. Precision will be inferred from the width of CIs around pooled estimates and the degree of uncertainty in reported metrics. Finally, publication bias will be examined using funnel plots and the Egger test, where applicable [57].

In addition to these core domains, AI-specific domains will further shape the evidence strength rating. Transparency of reporting, based on the TRIPOD-AI checklist, will be considered low if critical aspects of model development, validation, or data handling are rated as partially reported or not reported. Inadequate transparency may limit the interpretability and trustworthiness of findings and will result in downgrading the evidence.

### Transparency and Reproducibility

To support transparency and reproducibility, all data extraction forms, extracted study-level data, analysis code, and synthesis scripts developed for this review will be made openly available at the time of publication of the final results. Data extraction forms and extracted study-level data will be shared as multimedia appendices alongside the final publication. Analysis code and synthesis scripts, including those used for meta-analytic modeling, subgroup analyses, meta-regression, and figure generation, will be deposited in a public Zenodo repository, with the corresponding DOI reported in the final publication. Reproducibility findings derived from the NeurIPS paper checklist will be summarized narratively in the *Results* section, highlighting patterns in data and code availability, licensing, and reporting completeness across included studies.

## Results

**Figure 1.**
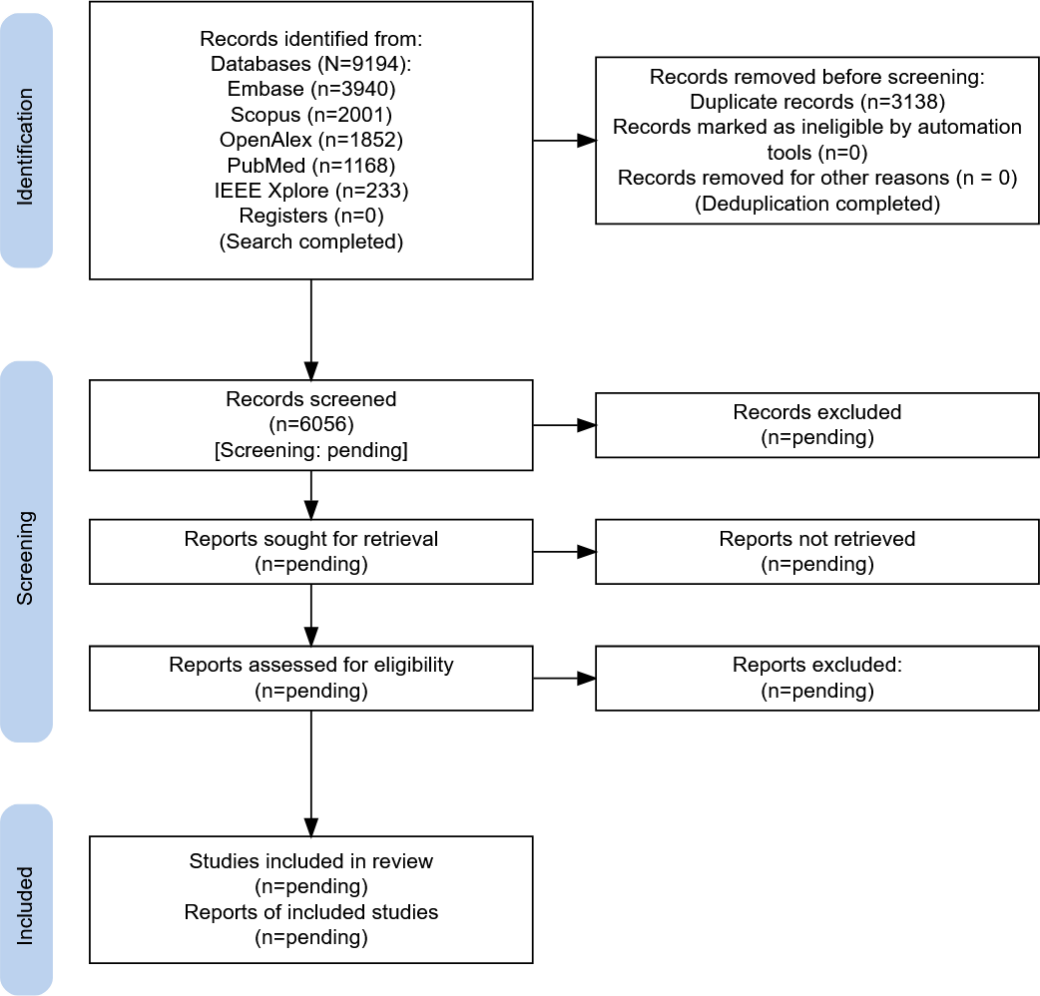
Preferred Reporting Items for Systematic Reviews and Meta-Analyses (PRISMA) 2020 flow diagram of the study selection process for the systematic review and meta-analysis of AI-based prognostic models among people living with HIV.

The literature search was completed in June 2026, yielding a total of 9194 records across the 5 bibliographic databases. Following automated deduplication, 3138 duplicate records were removed, leaving 6056 unique records pending title and abstract screening (Figure 1). Full-text eligibility assessment, data extraction, quality appraisal, and quantitative synthesis are expected to begin in August 2026, with the final results expected to be published by late 2026.

Upon completion, the review is expected to identify a diverse body of AI-based prognostic models applied across key stages of the HIV care continuum. Some models may demonstrate moderate to high discriminative performance. However, pooled AUC estimates will be interpreted with caution given the anticipated predominance of internally validated models and the limited availability of external validation evidence. Moreover, substantial heterogeneity is expected owing to variations in outcome definitions, modeling techniques, data sources, and validation strategies. The review is also likely to reveal frequent methodological and reporting limitations, including gaps in reporting transparency, reproducibility, and RoB.

## Discussion

This protocol outlines a rigorous and comprehensive approach to systematically reviewing AI-based prognostic models applied to the care of people living with HIV (Multimedia Appendix 2). Recent evidence, including the global systematic review by Ngcobo et al [29], has demonstrated the rapid expansion of AI applications across the HIV care continuum, encompassing diagnostics, testing, retention, treatment monitoring, and patient support. However, that synthesis adopted a broad narrative mapping approach and did not specifically evaluate AI-based prognostic prediction models or quantitatively assess predictive performance, RoB, reporting quality, or readiness for clinical implementation.

Building on this foundation, this protocol is designed to address the unresolved gap by exclusively considering individual-level AI-based prognostic models that predict treatment and disease outcomes among people living with HIV. By focusing on model objectives, predictors, data sources, algorithm types, performance metrics, and methodological rigor, this review will provide a synthesis of AI applications in HIV prognosis. The inclusion of both ML and deep learning models broadens the scope and ensures that emerging technologies are critically appraised alongside traditional approaches.

The methodological choices for this review, including the use of standardized data extraction instruments and multitool appraisal strategies (CHARMS, PROBAST+AI, TRIPOD-AI, DECIDE-AI, and NeurIPS checklists), are intended to move beyond descriptive cataloging toward a structured evaluation of model validity, transparency, and translational potential. PROBAST+AI will ensure a systematic appraisal of both the model development and evaluation phases, with attention to sources of bias, applicability, and analytical robustness, while also addressing AI-specific concerns such as algorithmic fairness, data leakage, class imbalance, and overfitting. The integrated appraisal framework will therefore enable the simultaneous assessment of methodological rigor, reporting completeness, reproducibility, and clinical relevance—dimensions not comprehensively examined in prior HIV-focused AI reviews.

In addition to model-level appraisal, the protocol incorporates an assessment of the overall strength of the body of evidence, adapted from GRADE principles and extended to reflect AI-specific considerations. This extension is particularly important given the high prevalence of internally validated models, limited external validation, and inconsistent reporting observed in the existing literature. Evaluating consistency, precision, directness, and potential publication bias will provide a nuanced understanding of the confidence that can be placed in the synthesized findings. The combined use of DECIDE-AI and the NeurIPS paper checklist will further evaluate real-world implementation barriers, system integration challenges, user interaction issues, and reproducibility constraints—factors that are often underreported yet critical for translation into clinical practice.

The anticipated meta-analysis of prognostic accuracy metrics, especially using diagnostic test accuracy methods, is an adaptation that underscores the complexity of evaluating AI model performance across heterogeneous studies. Unlike prior narrative syntheses, this protocol clearly conditions the meta-analysis on clinical and methodological comparability, with narrative synthesis retained as the primary analytical approach where heterogeneity precludes meaningful pooling. The planned use of random-effects models, HSROC curves, and meta-regression will allow the exploration of heterogeneity related to algorithm classes, validation strategies, outcome types, and population characteristics, thereby identifying factors that systematically influence prognostic performance.

The review will also address methodological diversity in prediction model design, including dynamic and time-updated prognostic models that revise predictions in response to evolving patient data, such as longitudinal viral load trajectories, serial CD4 measurements, or treatment history. These models, often implemented using recurrent architectures, such as RNNs or LSTMs, or through landmark modeling approaches, predict not only whether a clinical event may occur but also when it will occur and are relevant to HIV care, given the longitudinal nature of ART monitoring. When such models are identified, their dynamic updating mechanisms, landmark time points, and time-horizon specifications will be captured during data extraction and reported as a distinct methodological subgroup in the narrative synthesis [58,59].

Ultimately, this review will offer a comprehensive overview of the current landscape of AI-driven prognostic modeling in HIV care. It complements recent broad reviews of AI in HIV care by providing a more in-depth, prediction-focused synthesis that clarifies which AI-based prognostic models are methodologically sound, clinically applicable, and ready for further evaluation or implementation. Through the systematic identification of persistent gaps in reporting, validation, and implementation, and the mapping of intended clinical uses, such as decision support, treatment monitoring, and resource allocation, this review aims to help bridge the gap between algorithm development and real-world HIV care. Such alignment may contribute to informing precision medicine approaches and, where models are adequately validated, supporting improved long-term outcomes for people living with HIV.

## Supplementary material

10.2196/91989 Multimedia Appendix 1 Search terms used to retrieve publications on AI, HIV and outcomes.

10.2196/91989 Multimedia Appendix 2 A visual abstract summarizing the protocol of the research titled "AI for prognosis among people living with HIV: a systematic review and meta-analysis protocol," published in JMIR Research Protocols in 2026.

10.2196/91989 Checklist 1 PRISMA-P checklist.

## Abbreviations

AHD: advanced HIV disease
ART: antiretroviral therapy
AUC: area under the curve
CD4: cluster of differentiation 4
CHARMS: Checklist for Critical Appraisal and Data Extraction for Systematic Reviews of Prediction Modelling Studies
DECIDE-AI: Developmental and Exploratory Clinical Investigations of Decision Support Systems Driven by AI
GRADE: Grading of Recommendations Assessment, Development, and Evaluation
HSROC: hierarchical summary receiver operating characteristic
LSTM: long short-term memory
ML: machine learning
NeurIPS: Neural Information Processing Systems
PRISMA-P: Preferred Reporting Items for Systematic Reviews and Meta-Analyses Protocols
PROBAST: Prediction Model Risk of Bias Assessment Tool
PROBAST+AI: Prediction Model Risk of Bias Assessment Tool Extension for AI
PROSPERO: Prospective Register of Systematic Reviews
RNN: recurrent neural network
RoB: risk of bias
TRIPOD-AI: Transparent Reporting of Multivariable Prediction Models for Individual Prognosis or Diagnosis Extension for AI
TRIPOD-SRMA: Transparent Reporting of Multivariable Prediction Models for Individual Prognosis or Diagnosis: Checklist for Systematic Reviews and Meta-Analyses
