# Supplementary material for: AI for Prognosis Among People Living With HIV: Protocol for a Systematic Review and Meta-Analysis
Source: JMIR Res Protoc. 2026 Aug 18;15:e91989. doi: 10.2196/91989 (PMC13484944; doi:10.2196/91989)
Supplement: Supplementary file 1 [file resprot-v15-e91989-s001.pdf]

Table S1. Search terms used to retrieve publications on AI, HIV and outcomes

Database	Search Terms	Number of Records
PubMed	(((Artificial Intelligence[MeSH]) OR (Machine Learning[MeSH]) OR (Deep Learning[MeSH]) OR (Neural Networks, Computer[MeSH]) OR (Classification Algorithms[MeSH]) OR (artificial intelligence[Title/Abstract]) OR (machine learning[Title/Abstract]) OR (classification algorithm[Title/Abstract]) OR (decision support[Title/Abstract]) OR (rule-based system[Title/Abstract]) OR (Supervised Machine Learning[Mesh]) OR (Supervised learning[Title/Abstract]) OR (Automated system[Title/Abstract]) OR (digital health[Title/Abstract]) OR (Health informatics[Title/Abstract]))<br>AND<br>((HIV[MeSH]) OR (Acquired Immunodeficiency Syndrome[MeSH]) OR (HIV[Title/Abstract]) OR (HIV/AIDS[Title/Abstract]) OR (human immunodeficiency virus[Title/Abstract]) OR (human immune deficiency virus[Title/Abstract]) OR (human immuno-deficiency virus[Title/Abstract]) OR (human immune-deficiency virus[Title/Abstract]) OR (acquired immunodeficiency syndrome[Title/Abstract]) OR (acquired immune deficiency syndrome[Title/Abstract]) OR (acquired immuno-deficiency syndrome[Title/Abstract]) OR (acquired immune-deficiency syndrome[Title/Abstract]) OR (advanced HIV disease[Title/Abstract]) OR (people living with HIV[Title/Abstract]) OR (PLHIV[Title/Abstract]))<br>AND<br>((suppression[Title/Abstract]) OR (failure[Title/Abstract]) OR (rebound[Title/Abstract]) OR (load[Title/Abstract]) OR (HIV RNA[Title/Abstract]) OR (CD4[Title/Abstract]) OR (reconstitution[Title/Abstract]) OR (progression[Title/Abstract]) OR (opportunistic[Title/Abstract]) OR (mortality[Title/Abstract]) OR (death[Title/Abstract]) OR (survival[Title/Abstract]) OR (hospitalization[Title/Abstract]) OR (hospitalisation[Title/Abstract]) OR (inpatient[Title/Abstract]) OR (discontinuation[Title/Abstract]) OR (resistance[Title/Abstract]) OR (retention[Title/Abstract]) OR (follow-up[Title/Abstract]) OR (engagement[Title/Abstract]) OR (disengagement[Title/Abstract]) OR (adherence[Title/Abstract]) OR (attendance[Title/Abstract]) OR (visit[Title/Abstract]) OR (linkage[Title/Abstract]) OR (continuum[Title/Abstract]) OR (cascade[Title/Abstract]) OR (comorbidity[Title/Abstract]) OR (cardiovascular[Title/Abstract]) OR (renal[Title/Abstract]) OR (kidney[Title/Abstract]) OR (metabolic[Title/Abstract]) OR (mental[Title/Abstract]) OR (psych*[Title/Abstract]) OR (neuro*[Title/Abstract]) OR (depression[Title/Abstract]) OR (weight loss[Title/Abstract]) OR (body weight[Title/Abstract]) OR (wasting[Title/Abstract]) OR (malnutrition[Title/Abstract]) OR (anaemia[Title/Abstract]) OR (anemia[Title/Abstract]) OR (tuberculosis[Title/Abstract]) OR (coinfection[Title/Abstract]) OR (hepatitis[Title/Abstract]) OR (toxicity[Title/Abstract]) OR (effect[Title/Abstract]) OR (prognosis[Title/Abstract]) OR (prognostic[Title/Abstract]) OR (outcome[Title/Abstract])))<br><br>Filter: Year: 2015 – 2025	1,168
Embase	('artificial intelligence'/exp OR 'machine learning'/exp OR 'deep learning'/exp OR 'neural network'/exp OR 'classification algorithm'/exp OR 'supervised learning'/exp OR 'artificial intelligence':ti,ab OR 'machine learning':ti,ab OR 'classification algorithm':ti,ab OR 'decision support':ti,ab OR 'rule-based	3,940

	system':ti,ab OR 'supervised learning':ti,ab OR 'automated system':ti,ab OR 'digital health':ti,ab OR 'health informatics':ti,ab)<br>AND<br>('hiv'/exp OR 'acquired immune deficiency syndrome'/exp OR 'hiv':ti,ab OR 'hiv/aids':ti,ab OR 'human immunodeficiency virus':ti,ab OR 'human immune deficiency virus':ti,ab OR 'human immuno-deficiency virus':ti,ab OR 'human immune-deficiency virus':ti,ab OR 'acquired immunodeficiency syndrome':ti,ab OR 'acquired immune deficiency syndrome':ti,ab OR 'acquired immuno-deficiency syndrome':ti,ab OR 'acquired immune-deficiency syndrome':ti,ab OR 'advanced hiv disease':ti,ab OR 'people living with hiv':ti,ab OR 'plhiv':ti,ab)<br>AND<br>(suppression:ti,ab OR failure:ti,ab OR rebound:ti,ab OR load:ti,ab OR 'hiv rna':ti,ab OR 'cd4':ti,ab OR reconstitution:ti,ab OR progression:ti,ab OR opportunistic:ti,ab OR mortality:ti,ab OR death:ti,ab OR survival:ti,ab OR hospitalization:ti,ab OR hospitalisation:ti,ab OR inpatient:ti,ab OR discontinuation:ti,ab OR resistance:ti,ab OR retention:ti,ab OR 'follow-up':ti,ab OR engagement:ti,ab OR disengagement:ti,ab OR adherence:ti,ab OR attendance:ti,ab OR visit:ti,ab OR linkage:ti,ab OR continuum:ti,ab OR cascade:ti,ab OR comorbidity:ti,ab OR cardiovascular:ti,ab OR renal:ti,ab OR kidney:ti,ab OR metabolic:ti,ab OR mental:ti,ab OR psych*:ti,ab OR neuro*:ti,ab OR depression:ti,ab OR 'weight loss':ti,ab OR 'body weight':ti,ab OR wasting:ti,ab OR malnutrition:ti,ab OR anaemia:ti,ab OR anemia:ti,ab OR tuberculosis:ti,ab OR coinfection:ti,ab OR hepatitis:ti,ab OR toxicity:ti,ab OR effect:ti,ab OR prognosis:ti,ab OR prognostic:ti,ab OR outcome:ti,ab)<br><br>Filters:<br>Year: 2015 – 2025<br>Publication type: Article	
Scopus	((TITLE-ABS-KEY("artificial intelligence") OR TITLE-ABS-KEY("machine learning") OR<br>TITLE-ABS-KEY("deep learning") OR TITLE-ABS-KEY("neural network*") OR<br>TITLE-ABS-KEY("classification algorithm") OR TITLE-ABS-KEY("decision support") OR<br>TITLE-ABS-KEY("rule-based system") OR TITLE-ABS-KEY("supervised learning") OR<br>TITLE-ABS-KEY("automated system") OR TITLE-ABS-KEY("digital health") OR<br>TITLE-ABS-KEY("health informatics"))<br>AND<br>(TITLE-ABS-KEY("HIV") OR TITLE-ABS-KEY("HIV/AIDS") OR<br>TITLE-ABS-KEY("human immunodeficiency virus") OR<br>TITLE-ABS-KEY("human immune deficiency virus") OR<br>TITLE-ABS-KEY("human immuno-deficiency virus") OR<br>TITLE-ABS-KEY("human immune-deficiency virus") OR<br>TITLE-ABS-KEY("acquired immunodeficiency syndrome") OR<br>TITLE-ABS-KEY("acquired immune deficiency syndrome") OR<br>TITLE-ABS-KEY("acquired immuno-deficiency syndrome") OR<br>TITLE-ABS-KEY("acquired immune-deficiency syndrome") OR<br>TITLE-ABS-KEY("advanced HIV disease") OR<br>TITLE-ABS-KEY("people living with HIV") OR TITLE-ABS-KEY("PLHIV"))<br>AND<br>(TITLE-ABS-KEY(suppression) OR TITLE-ABS-KEY(failure) OR<br>TITLE-ABS-KEY(rebound) OR TITLE-ABS-KEY(load) OR<br>TITLE-ABS-KEY("HIV RNA") OR TITLE-ABS-KEY("CD4") OR<br>TITLE-ABS-KEY(reconstitution) OR TITLE-ABS-KEY(progression) OR<br>TITLE-ABS-KEY(opportunistic) OR TITLE-ABS-KEY(mortality) OR	2,001

	TITLE-ABS-KEY(death) OR TITLE-ABS-KEY(survival) OR<br>TITLE-ABS-KEY(hospitalization) OR TITLE-ABS-KEY(hospitalisation) OR<br>TITLE-ABS-KEY(inpatient) OR TITLE-ABS-KEY(discontinuation) OR<br>TITLE-ABS-KEY(resistance) OR TITLE-ABS-KEY(retention) OR<br>TITLE-ABS-KEY("follow-up") OR TITLE-ABS-KEY(engagement) OR<br>TITLE-ABS-KEY(disengagement) OR TITLE-ABS-KEY(adherence) OR<br>TITLE-ABS-KEY(attendance) OR TITLE-ABS-KEY(visit) OR<br>TITLE-ABS-KEY(linkage) OR TITLE-ABS-KEY(continuum) OR<br>TITLE-ABS-KEY(cascade) OR TITLE-ABS-KEY(comorbidity) OR<br>TITLE-ABS-KEY(cardiovascular) OR TITLE-ABS-KEY(renal) OR<br>TITLE-ABS-KEY(kidney) OR TITLE-ABS-KEY(metabolic) OR<br>TITLE-ABS-KEY(mental) OR TITLE-ABS-KEY(psych*) OR<br>TITLE-ABS-KEY(neuro*) OR TITLE-ABS-KEY(depression) OR<br>TITLE-ABS-KEY("weight loss") OR TITLE-ABS-KEY("body weight") OR TITLE-ABS-KEY(wasting) OR TITLE-ABS-KEY(malnutrition) OR<br>TITLE-ABS-KEY(anaemia) OR TITLE-ABS-KEY(anemia) OR<br>TITLE-ABS-KEY(tuberculosis) OR TITLE-ABS-KEY(coinfection) OR<br>TITLE-ABS-KEY(hepatitis) OR TITLE-ABS-KEY(toxicity) OR<br>TITLE-ABS-KEY(effect) OR TITLE-ABS-KEY(prognosis) OR<br>TITLE-ABS-KEY(prognostic) OR TITLE-ABS-KEY(outcome)))<br>AND PUBYEAR > 2014 AND PUBYEAR < 2026<br>AND (DOCTYPE("ar") OR DOCTYPE("cp"))	
OpenAlex	("artificial intelligence" OR "machine learning" OR "deep learning" OR "neural network" OR "classification algorithm" OR "decision support" OR "rule-based system" OR "supervised learning" OR "automated system" OR "digital health" OR "health informatics")<br>AND<br>("HIV" OR "HIV/AIDS" OR "human immunodeficiency virus" OR "human immune deficiency virus" OR "human immuno-deficiency virus" OR "human immune-deficiency virus" OR "acquired immunodeficiency syndrome" OR "acquired immune deficiency syndrome" OR "advanced HIV disease" OR "people living with HIV" OR PLHIV)<br>AND<br>(suppression OR failure OR rebound OR load OR "HIV RNA" OR CD4 OR reconstitution OR progression OR opportunistic OR mortality OR death OR survival OR hospitalization OR hospitalisation OR inpatient OR discontinuation OR resistance OR retention OR "follow-up" OR engagement OR disengagement OR adherence OR attendance OR visit OR linkage OR continuum OR cascade OR comorbidity OR cardiovascular OR renal OR kidney OR metabolic OR mental OR psych* OR neuro* OR depression OR "weight loss" OR "body weight" OR wasting OR malnutrition OR anaemia OR anemia OR tuberculosis OR coinfection OR hepatitis OR toxicity OR effect OR prognosis OR prognostic OR outcome)<br><br>Filters:<br>Year: 2015 – 2025<br>Type: Article, Preprint	1,852
IEEE Xplore	("artificial intelligence" OR "machine learning" OR "deep learning" OR "neural network" OR "classification algorithm" OR "decision support" OR "rule-based system" OR "supervised learning" OR "automated system" OR "digital health" OR "health informatics") AND<br>("HIV" OR "HIV/AIDS" OR "human immunodeficiency virus" OR "human immune deficiency virus" OR "human immuno-deficiency virus" OR "human immune-deficiency virus" OR "acquired immunodeficiency syndrome" OR "acquired immune deficiency syndrome" OR "acquired immuno-deficiency syndrome" OR "acquired immune-deficiency syndrome" OR "advanced HIV disease" OR "people living with HIV" OR "PLHIV")<br>AND	233

	(suppression OR failure OR rebound OR load OR "HIV RNA" OR "CD4" OR reconstitution OR progression OR opportunistic OR mortality OR death OR survival OR hospitalization OR hospitalisation OR inpatient OR discontinuation OR resistance OR retention OR "follow-up" OR engagement OR disengagement OR adherence OR attendance OR visit OR linkage OR continuum OR cascade OR comorbidity OR cardiovascular OR renal OR kidney OR metabolic OR mental OR psych* OR neuro* OR depression OR "weight loss" OR "body weight" OR wasting OR malnutrition OR anaemia OR anemia OR tuberculosis OR coinfection OR hepatitis OR toxicity OR effect OR prognosis OR prognostic OR outcome)<br><br>Filters:<br>Year: 2015 – 2025<br>Publication type: Conferences, Journals	
